# Author response to: Routine *versus* selective intraoperative cholangiography during cholecystectomy: a systematic review, meta-analysis, and health economic model analysis of iatrogenic bile duct injury

**DOI:** 10.1093/bjsopen/zrac054

**Published:** 2022-04-13

**Authors:** Jenny M. L. Rystedt, Agneta K. Montgomery

**Affiliations:** Department of Surgery, Skane University Hospital, Clinical Sciences, Lund University, Lund, Sweden; Department of Surgery, Skane University Hospital, Clinical Sciences, Lund University, Lund, Sweden

*Dear Editor*

Indeed, a bile duct injury (BDI) can occur after a normal intraoperative cholangiography (IOC), but a roadmap with exact positioning of the catheter, in the operation field and on the ‘IOC-map’ simultaneously, strongly reduces the risk of anatomical misinterpretation and confirmation bias during dissection. The study concluded that routine IOC prevents seven BDIs annually in Sweden and increases the rate of intraoperative diagnosis, leading to reduced costs and better patient-reported quality of life^1^. As pointed out by Hung *et al*.^2^, the criteria for the strategy of selective IOC and the timing of IOC in relation to the BDI were not possible to analyse in the current meta-analysis, but the strategy of routine IOC was superior to the selective use in terms of BDI risk. In addition to preventing BDI and saving quality-adjusted life years, we advocate a routine use of IOC since selective use can be time-consuming for surgeons who are not familiar with the procedure.

Hung *et al*. suggest that indocyanine green fluorescence cholangiography (ICG) should replace IOC. We acknowledge that ICG has the benefit of no X-ray radiation, but ICG has also clear drawbacks as there is the need for planning and timing of the preoperative injection. Although many show interest in intraoperative imaging with ICG use in laparoscopic cholecystectomy, we believe that the present level of knowledge and scientific evidence are insufficient to declare it superior to IOC.
